# Difficulties in Practice

**Published:** 1809-07

**Authors:** 


### *To the Editors of the Medical and Physical Journal.*

Gentlemen,

In your Number, (116,) for October, a Correspondent who signs himself "A Constant Reader," has occupied the pages of your miscellany by some animadversions on a paper communicated by Mr. Carter. I shall not enquire whether the castigation be deserved or not, that I leave to Mr. C.; nor whether your "Constant Reader" would not do well (before he sends you another communication) to read his last, to ponder upon its import, and lay its counsels impartially to his own heart.—*Mutato nomine de te, &c.* For myself, I do not write to convey, but to solicit information, thinking that my communication may not be useless, if if it give rise to the observations of your well informed and experienced Correspondents, though in itself, it contains "nothing original," or that "every one at all conversant with the practice of medicine" is unacquainted with.

In the practice of an art so confessedly imperfect as that of medicine necessarily is, the best way to advance the object of our common ambition, seems to be that of collecting and concentrating the knowledge and experience of the practitioners of the art. The intelligent reader, possessed of a collection of facts, will for himself institute the comparison, will by deduction, fix upon that plan of practice which has been most generally successful, and keeping it in view, will introduce into it such occasional variety of practice, as may best meet the endless variety of disease. In this consists the immense advantage of the well-informed and reflecting practitioner, over the mere imitative empiric, whether regularly or irregularly educated to the profession. There is a general resemblance in certain diseases which we call by the same name, but no two cases, perhaps, ever occurred precisely alike, nor admitted of exactly similar practice. There is such a family likeness, that they are acknowledged to be of the same stock, but an accurate observer will see sufficient difference to prevent his identifying two members of one house. In adopting the modes of practice to this diversity, consist at once the difficulty and the glory of the medical art. The good practitioner must be as fertile in resources, as disease is fertile in symptoms, be as quick as is the progress of the complaint, and have his observation exerted as unceasingly as the complicated operations of nature, struggling with disease, continue to take place. Without pursuing these general observations into detail, I proceed to fulfil the object of this communication, to state certain difficulties in practice, and to solicit the attention, the knowledge, and the information of your Correspondents.

1. *Amenorrhœa.* I apprehend there are few practitioners who have not found themselves baffled in all their attempts to assist nature in the producing or restorating a periodical evacuation, intimately connected with the perfect health of the female. The difficulty attaches (though not with equal force) to (1) *emansio mensium*—(2) *suppressio mensium*, and to (3) *dysmenorrhœa.*

2. Leucorrhœa. The same difficulty and uncertainty in practice attach to this disease, whether in its *continued* or *occasional* form.

*Chlorosis,* till very lately, might have been added to the number, number, but the practice instituted by Dr. Hamilton, sen. of Edinburgh, and supported by the cases which that physician has published, has been so generally successful, that I forbear to add it. Not that the practice has (with me at least) generally succeeded in abolishing the "retentio mensium," so frequently a symptom of the disease, but all the other symptoms have yielded to the steady prosecution of the plan.

Here, for the present, I desist. If the idea which I have in this paper thrown out, meet the approbation and call forth the communications of your Correspondents, I shall from time to time resume the subject, state briefly the difficulty and uncertainty attending the treatment of many other diseases of daily occurrence, and hope to benefit the medical art by causing the result of the skill and experience of your Correspondents to be collected and recorded in your Journal. Facts are the only sure foundation on which the arts and sciences rest. To collect, to compare, to arrange, and to draw deductions from them, are the lessons and exercises which we have to learn in the school of practice. There is no other road to knowledge, and I hope it will gradually become a more public and beaten way.

